# Droplet Digital PCR (ddPCR) for Halal Detection of Commercial Gelatin Products

**DOI:** 10.1155/ijfo/8841767

**Published:** 2025-10-22

**Authors:** Noviyan Darmawan, Safira Gina, Cintera Rahmagiarti, Isna Mustafiatul Ummah, Cece Sumantri, Irma Herawati Suparto, Seagames Waluyo

**Affiliations:** ^1^ Department of Chemistry, IPB University, Bogor, Indonesia, ipb.ac.id; ^2^ Halal Science Center, IPB University, Bogor, Indonesia, ipb.ac.id; ^3^ Application Scientist Division, PT Sciencewerke, Jakarta, Indonesia; ^4^ Department of Animal Production and Technology, IPB University, Bogor, Indonesia, ipb.ac.id

**Keywords:** ddPCR, DNA, gelatin, halal, porcine

## Abstract

A droplet digital polymerase chain reaction (ddPCR) assay was developed for the identification and quantification of porcine DNA in commercial gelatin products. The method employed primers targeting the beta‐actin gene and demonstrated high specificity against five nontarget species across 18 different products. The ddPCR method showed enhanced sensitivity, detecting porcine DNA at concentrations as low as 0.06 ng/*μ*L in standard gelatin powder, surpassing the detection limit of 0.11 ng/*μ*L achieved by real‐time PCR. The robustness of the assay was validated using seven types of commercial porcine gelatin products, including candies, marshmallows, dietary supplements, collagen, and capsule shells. ddPCR analysis revealed that six out of seven commercial product samples tested positive for porcine DNA, compared to only three samples detected by RT‐PCR. Sequencing of the ddPCR amplicons further confirmed the accuracy of the assay, with high similarity to the reference porcine species.

## 1. Introduction

Commercial gelatin‐based products are extensively used in various sectors such as food, cosmetics, and pharmaceuticals every day by consumers. Recently, there has been a significant surge in the demand for halal gelatin due to the rapid and substantial growth observed in the global halal industry, coupled with heightened young consumer awareness regarding product halal status. Typically, one of the prerequisites for halal products is that their raw materials must not originate from porcine sources. However, the main materials utilized in large‐scale gelatin production include bones and skins derived from both bovine and porcine origins, thereby posing a significant risk of potential fraud, false labeling, and adulteration among these gelatin sources [1, 2]. Consequently, the development of reliable and sensitive halal detection methods aimed at differentiating and identifying the source of gelatin becomes required.

Various analytical techniques have been developed and documented to authenticate the origin of gelatin species. Among these, the real‐time polymerase chain reaction (PCR) (RT‐PCR) method has emerged as a widely employed approach, using specific primers designed for porcine DNA to assess DNA contamination within gelatin matrices. However, the efficacy of RT‐PCR analysis is limited by the minimal quantity of DNA and the significant level of DNA denaturation within gelatin matrices, especially in highly processed gelatin products. On the other hand, the droplet digital PCR (ddPCR) method has emerged as a promising tool for food authentication, offering enhanced precision and sensitivity in quantifying target DNA at low concentrations without requiring a standard curve as in RT‐PCR. In principle, this technique involves partitioning the amplification reaction mixture into numerous droplets, enabling the accurate determination of target DNA copies through positive and negative droplet counts using the Poisson algorithm. Ampaporn et al. [3] have successfully utilized ddPCR to quantify porcine DNA in processed meat products, achieving a detection limit of 0.0001 ng. While Nuraeni et al. [4] showed the superior sensitivity of ddPCR over RT‐PCR in detecting porcine DNA within plasmids, revealing a detection limit of 1 copy per reaction as opposed to 5 copies per reaction for RT‐PCR. Furthermore, Mahamad et al. [5] have demonstrated the utility of the ddPCR method in detecting porcine gelatin within capsule products, exhibiting high detection limits and specificity. However, no reports have explored the application of ddPCR for identifying porcine gelatin in other commercial products like highly processed food and cosmetics. Unlike pharmaceutical capsules, these products possess a more complex matrix due to their extensive production processes and incorporation of various substances such as preservatives, flavors, spices, and other compounds, resulting in a lower amount of highly degraded porcine DNA contamination within gelatin matrices.

Recently, we have shown that the choice of a commercial DNA extraction kit significantly influences the successful extraction of DNA from commercial gelatin product matrices, such as candies, consequently impacting the efficacy of PCR analysis [6]. Hence, the objective of this study is to develop a halal detection technique using ddPCR methods for identifying and quantifying porcine DNA in commercial porcine gelatin–based products, namely, candies, marshmallows, health supplements, collagen, and capsule shells. We exclusively selected products labeled as containing porcine gelatin, and the performance of this ddPCR method, encompassing specificity, detection limit, and robustness, will be assessed and compared to that of the RT‐PCR method using identical samples.

## 2. Materials and Methods

### 2.1. Materials

Porcine gelatin powder (Sigma‐Aldrich, United States) was used as a reference standard for optimizing the ddPCR method. Processed food products containing porcine gelatin, such as capsule shells, dietary supplements, marshmallows, collagen, milk candy, soft candy, and gummy candy, as well as beef meat, fish meat, rabbit meat, shrimp meat, chicken sausage, beef gelatin, fish gelatin, and pork meat, were purchased from the commercial market. The extraction kits used were the Processed Food DNA Extraction Kit (TIANGEN, China) and the ProgenusEasyFast Pharma I Kit (Belgium).

### 2.2. DNA Extraction

Prior to DNA extraction, capsule shells, candy, marshmallows, and meat were sectioned into small pieces using sterile scissors. DNA extraction from porcine gelatin powder, fresh meat, chicken sausage, candy, marshmallows, collagen, and dietary supplements was performed using the Processed Food DNA Extraction Kit with modifications (TIANGEN, China). For capsule shells, the ProgenusEasyFast Pharma I Kit (Belgium) was utilized, following the protocol with some modifications. The concentration and purity of the extracted DNA were measured using a MaestroNano spectrophotometer (MaestroGen, China), and the extracted DNA was stored at −20°C for subsequent analysis.

### 2.3. Primer and Probe

Primers and probes targeting the *Sus scrofa–*specific beta‐actin (ACTB) gene were employed for the detection of porcine DNA, with the GenBank accession number DQ452569.1 (NCBI). The primer and TaqMan probe designs, adhering to ISO/TS 20224‐3:2020(E), are detailed in Table 1.

**Table 1:** Primer and probe sequences used in this study.

**Primer/probe**	**Oligonucleotide DNA sequences**
Porcine‐97bp‐F	5 ^′^‐ CGTAGGTGCACAGTAGGTCTGAC‐3 ^′^
Porcine‐97bp‐R	5 ^′^‐ GGCCAGACTGGGGACATG‐3 ^′^
Porcine‐97bp‐P	5 ^′^‐[FAM]‐CCAGGTCGGGGAGTC‐[MGBEclipse]‐3 ^′^
a. PCR product = 335 ‐ CGTAGG TGCACAGTAG GTCTGACGTG ACTCCCCGAC CTGGGGTCCC CAGCACACTT AGCCGTGTTC CTTGCACTCT CTGCATGTCC CCAGTCTGGC C ‐ 431 ‐ DQ452569.1 b. FAM: 6‐carboxyfluorescein, MGB: minor groove binder (nonfluorescent chromophore)	

### 2.4. ddPCR Procedure

The ddPCR procedure involved preparing a total reaction volume of 20 *μ*L, consisting of 10 *μ*L of ddPCR Supermix for Probes (no dUTP) (Bio‐Rad), 4 *μ*L of DNA template, 1.8 *μ*L of forward and reverse primers (final concentration 900 nM each), 0.5 *μ*L of TaqMan MGB Probe (final concentration 250 nM), 1 *μ*L of HindIII, and 0.9 *μ*L of nuclease‐free water (TIANGEN). The reaction mixture was loaded into a DG8 cartridge along with 70 *μ*L of droplet generation oil and processed in the QX200 Droplet Generator machine (Bio‐Rad) to generate droplets. These droplets were subsequently transferred into a 96‐well plate for PCR thermal cycling. The cycling conditions in the T100 Thermal Cycler (Bio‐Rad) included an initial denaturation step at 95°C for 10 min, followed by 40 cycles of denaturation at 94°C for 30 s, annealing–extension at 55°C for 60 s, enzyme deactivation at 98°C for 10 min, and final incubation at 4°C. The entire PCR protocol was conducted at a ramp rate of 2°C/s. Signal amplification and DNA copy distribution analysis were performed using a QX200 ddPCR Droplet Reader (Bio‐Rad).

### 2.5. Sensitivity Test

The limit of detection (LOD) was determined by diluting extracted DNA solutions from standard porcine gelatin, ranging from 3.43 to 0.03 ng/*μ*L. Each experiment was replicated, three replications for ddPCR and two replications for RT‐PCR.

### 2.6. Specificity Test

The specificity analysis was conducted on commercially purchased target and nontarget samples. Target DNA–containing samples included porcine gelatin and pork meat, whereas nontarget DNA samples comprised beef, fish, rabbit, shrimp, chicken sausage, beef gelatin, and fish gelatin. Double‐distilled water served as the negative control. The study was performed with two replications.

### 2.7. Robustness Test

The robustness testing involved seven commercially available porcine gelatin–based products, including marshmallows, three types of candies, dietary supplements, collagen, and capsule shells sourced from supermarkets in Indonesia. Each sample, weighing 200 mg, underwent DNA extraction. The extracted DNA, quantified for concentration and purity, was subsequently analyzed using the ddPCR method. The tests were conducted with two replications for ddPCR and four replications for RT‐PCR.

### 2.8. RT‐PCR Procedure

The RT‐PCR amplification was conducted using 20 *μ*L of each reaction solution run at CFX96 Real‐Time PCR (Bio‐Rad). The PCR reaction mixture consisted of 10 *μ*L iTaq Universal Probe Supermix (Bio‐Rad), 4 *μ*L DNA template, 0.8 *μ*L forward and reverse primers (final concentration 400 nM each), 0.4 *μ*L TaqMan MGB Probe (final concentration 200 nM), and 4 *μ*L nuclease‐free water (TIANGEN). The RT‐PCR method followed the ISO/TS 20224‐3:2020(E) protocol with initial denaturation at 95°C for 10 min, followed by 45 cycles of denaturation at 94°C for 15 s and annealing–extension at 60°C for 60 s. Data resulting from the RT‐PCR method were analyzed using the CFX Maestro 2.0 software (Bio‐Rad).

### 2.9. Amplicon Recovery

This test is done to verify the amplified DNA sequence in pig DNA. The steps are to pipette all the droplet and oil volumes from the ddPCR plate into a sterile 1.5‐mL tube. Then, pipette and discard the oil layer under the tube after the droplet layer rises to the top layer. Then, add 20 *μ*L TE buffer/well and then add 70 *μ*L/well chloroform and vortex for 1 min. Then, centrifuge at a speed of 15,500 × *g* for 10 min. The aqueous layer that forms (top side) is transferred with a micropipette to a new 1.5‐mL tube as part of the recovered DNA, and the chloroform layer was discarded. The DNA obtained was then sequenced by Macrogen using primers designed according to ISO/TS 20224‐3:2020(E).

### 2.10. Bioinformatics Analysis

The analysis was carried out using BioEdit and the Basic Local Alignment Search Tool (BLAST) [7, 8]. In the BioEdit software, an overview of the sequencing results from the recovery droplet on the gelatin sample will be obtained. Then, cut the sequence at 25 bp at the front and back and then analyze the similarity between the target sequence and the sequencing results. The results are also compared with those in BLAST between the gelatin sample and the reference and then align with those in the NCBI database, and the similarity value between the reference target and the gelatin sample will be obtained.

## 3. Result and Discussion

### 3.1. Concentration and Purity of DNA Extract

The extraction of DNA from gelatin to achieve high concentration and purity is essential for reliable PCR analysis [9]. A significant challenge in this process arises from the inherently low DNA content within gelatin matrices, where DNA often undergoes considerable degradation during manufacturing processes involving heat, acid, or base treatments [10]. Our previous research has demonstrated that the selection of the commercial extraction kit is critical in determining the concentration and purity of the extracted DNA [6]. In this study, the Processed Food DNA Extraction Kit was employed for the extraction of DNA from meat, candy, marshmallow, dietary supplements, and collagen, while the EasyFast Pharma I extraction kit was used for drug capsule shells. The concentrations of the DNA extracts ranged from 13.37 to 34.03 ng/*μ*L for gelatin powder, 0.69 to 623.58 ng/*μ*L for commercial products, and 79.83 to 228.11 ng/*μ*L for meat samples. The low DNA concentrations observed in capsule shells and marshmallows can be attributed to the complex sample matrices, which include numerous additives and diverse processing techniques [11]. Variations in DNA concentrations across other commercial products are likely due to differences in acid or alkaline treatments and manufacturing conditions.

DNA purity is another crucial factor for successful PCR amplification. The primary challenge in obtaining highly pure DNA was the effective separation of DNA from gelatin and other complex commercial product matrices because they contain additives such as sucrose syrup, acidifying agents, aromas, and food colorings [12, 13]. The purity of the extracted DNA was evaluated using a UV‐vis spectrophotometer, measuring the absorbance ratios at 260 and 280 nm (A260/A280). DNA extracts with A260/A280 ratios between 1.7 and 2.0 were deemed to be of high quality and suitable for PCR [14]. In this study, the A260/A280 purity ratios ranged from 1.95 to 2.11 for gelatin powder, 1.68 to 2.33 for commercial products, and 0.80 to 2.11 for meat samples (Table 2). Lower A260/A280 ratios indicated potential protein contamination [15]. Additionally, DNA purity was further assessed by measuring the absorbance ratio at 230 nm (A260/A230), which is indicative of the presence of organic compounds or chaotropic salts. A good A260/A230 ratio exceeds 1.5 [15]. The A260/A230 ratios reported in this study ranged from 1.87 to 2.12 for gelatin powder, 0.85 to 2.20 for commercial products, and 1.31 to 2.71 for meat samples. Differences in sample matrix composition and the presence of inhibitory such as heme might be the reason for the lesser purity of DNA extracted from meat samples [16]. Many commercial DNA extraction protocols optimized for processed food products such as gelatin may not work efficiently with complex raw matrices such as fresh meat as shown by Köppel et al. [17].

**Table 2:** DNA quality from different samples.

**Sample type**	**Concentration DNA (ng/*μ*L)**	**260/280**	**260/230**
Bovine gelatin	29.13	2.05	1.87
Porcine gelatin	34.03	1.95	2.12
Fish gelatin	13.37	2.11	1.94
Soft candy	286.07	2.33	0.85
Milk candy	32.24	1.82	2.20
Gummy candy	64.86	1.91	2.01
Marshmallow	0.69	1.80	2.08
Dietary supplement	623.58	1.50	1.44
Capsule shell	9.65	1.89	2.11
Collagen cosmetic	894.54	1.68	1.35
Pork meat	155.00	1.89	2.05
Beef meat	79.83	2.11	1.99
Fish meat	251.27	0.99	1.87
Rabbit meat	179.77	0.80	2.71
Shrimp meat	115.72	0.79	2.06
Chicken sausage	228.11	0.85	1.31

### 3.2. Sensitivity of ddPCR

**Figure 1:**
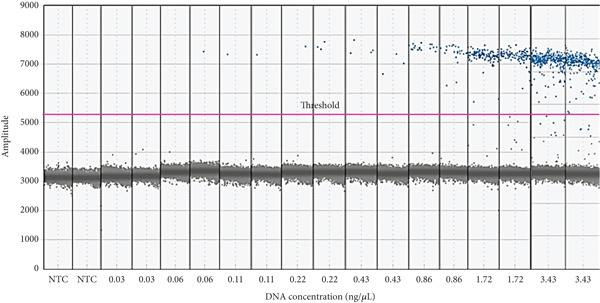
Sensitivity results of the ddPCR method which was carried out with a dilution DNA concentration of 3.43 ng/*μ*L up to 0.03 ng/*μ*L.

**Figure 2:**
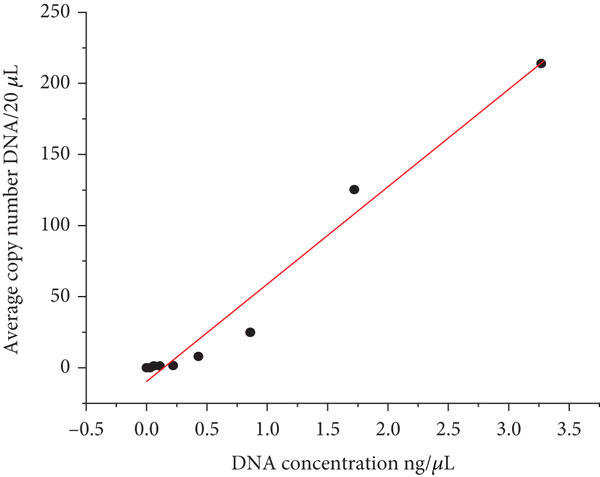
Linear regression correlation between DNA concentration and DNA copy number.

Further analysis was conducted to establish the linear relationship between DNA concentration and species‐specific target DNA copy number. This was achieved by diluting DNA extracts from gelatin powder samples and performing nontemplate control (NTC) analysis using ddPCR. To evaluate the sensitivity of the ddPCR method, half‐fold serial dilutions of gelatin powder DNA extracts were prepared, ranging from 3.43 to 0.03 ng/*μ*L. The LOD of the ddPCR assay was defined as the lowest DNA concentration with a coefficient of variation (CV) below 25% [18]. As illustrated in Figure 1, the LOD for the ddPCR assay was determined to be 0.06 ng/*μ*L, corresponding to 1.2 DNA copies per reaction. The ddPCR process generated at least 10,000 droplets per reaction, which met the criteria for absolute quantification. Fluorescence amplitude data showed that no positive droplets formed above the threshold line in the negative control, while a distinct separation was observed between negative and positive droplets (Figure 1). The correlation coefficient (*R* ^2^) between DNA concentration and DNA copy number was 0.973 (Figure 2), indicating a near‐linear relationship between concentration and DNA copy number. These findings suggested a consistent and reliable relationship between DNA concentration and target DNA copy number [3].

The efficiency observed in this study could be attributed to the primer design, which adhered to ISO/TS 20224‐3:2020(E) standards, targeting the ACTB gene. According to Ren et al. [19], the ACTB gene was superior to mitochondrial DNA (mtDNA) as a target because its haploid copy number remained constant, ensuring that DNA concentration was proportional to the number of samples tested. The ACTB gene provided more reliable predictions compared to mtDNA, whose copy number varied significantly across different organs and individuals. The high copy number of mtDNA could lead to uncertainty in quantification and reduced measurement accuracy [4]. This study also demonstrated that the ddPCR method exhibited greater sensitivity than RT‐PCR, with an LOD of 0.11 ng/*μ*L (Table 3). Nuraeni et al. [4] reported similar findings, showing that the ddPCR method could detect as low as 1 copy/reaction of porcine DNA plasmid, whereas the RT‐PCR method had a detection limit of 5 copies/reaction with a Cq value of 37. Additionally, Cao et al. [20] compared the performance of RT‐PCR and ddPCR in detecting silver pomfret fish DNA, finding that the ddPCR method was 156 times more sensitive, with an LOD of 2 copies/*μ*L compared to 313 copies/*μ*L for RT‐PCR. Wang et al. [21] also demonstrated that the ddPCR method for detecting *Salmonella typhimurium* was 10 times more sensitive, with an LOD of 10^−4^ ng/*μ*L compared to 10^−3^ ng/*μ*L for the RT‐PCR method. These results underscored the consistency and high sensitivity of the ddPCR method for detecting DNA at low concentrations across various test samples.

**Table 3:** Detection limits of ddPCR and RT‐PCR methods.

**DNA concentration (ng/*μ*L)**	**ddPCR**	**RT-PCR**
	**Average copy number (copy/reaction)**	**Average Cq value**
3.43	427 ± 0.21	28.61 ± 0.00
1.72	125.33 ± 0.25	30.07 ± 0.09
0.86	24.93 ± 0.19	32.62 ± 0.11
0.43	7.93 ± 0.34	34.58 ± 0.61
0.22	1.53 ± 0.09	36.03 ± 0.71
0.11	1.2 ± 0.00	37.37 ± 0.06
0.06	1.2 ± 0.00	NA
0.03	0 ± 0.00	NA

Previous studies, such as those conducted by Mahamad et al. [5], successfully detected bovine and porcine DNA in gelatin capsule using the ddPCR method. The detection limit found was 0.001 ng/*μ*L for porcine samples and 0.01 ng/*μ*L for bovine samples. The detection limit results differ from this study due to variability in DNA concentration values due to differences in the DNA content of the gelatin powder samples analyzed.

### 3.3. Specificity of ddPCR

**Figure 3:**
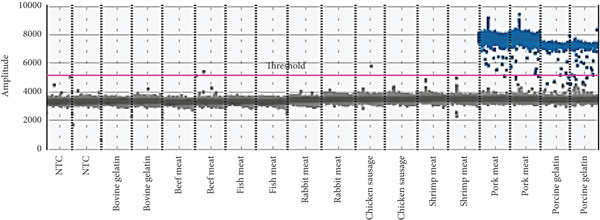
Visualization of positive and negative droplet populations of specificity with the ddPCR method.

Specificity was a fundamental requirement for both qualitative and quantitative methods in food authentication. To evaluate the specificity of the ddPCR assay for porcine species, samples from five nontarget animal species—bovine, fish, rabbit, shrimp, and chicken—were tested. The species‐specific primers and probes selectively amplified the target sequence from porcine DNA, with no cross‐reactivity observed in the nontarget species (Figure 3). These findings confirmed that the ddPCR assay accurately identified porcine species. The primers and probes, designed in accordance with ISO/TS 20224‐3:2020, were proven to specifically detect domestic pigs (*Sus scrofa domesticus*) and wild boars (*Sus scrofa*), while yielding negative results for nontarget DNA. The specificity of the assay was further validated by its ability to selectively detect porcine DNA in pork meat and porcine gelatin, with copy numbers of 7060 and 214 per reaction, respectively (Figure 3).

Mahamad et al. [5] similarly reported no cross‐reactivity in ddPCR tests using primers and probes specifically designed for porcine and bovine DNA against a range of nontarget species, including goats, sheep, dogs, deer, shellfish, frogs, squid, salmon, tuna, shrimp, duck, and chicken. The use of primers for pork meat in ddPCR was also confirmed by Ampaporn et al. [3], who demonstrated that the assay was selective for pork meat at a target concentration of 11.90 copies/*μ*L. Additionally, Shehata et al. [22] demonstrated the effectiveness of ddPCR in detecting DNA from cattle, pork, chicken, and turkey in raw meat, processed products, and feed. Floren et al. [18] further validated the ability of ddPCR to accurately detect DNA from horses, cattle, and pigs. Collectively, these studies established ddPCR as a robust tool for the selective detection of porcine DNA in gelatin‐containing products.

### 3.4. Robustness of ddPCR

The robustness of the developed ddPCR method was evaluated by detecting porcine DNA in seven commercial gelatin products, including three types of candy, marshmallows, health supplements, capsule shells, and collagen (Table 4). These products were labeled as containing porcine gelatin and no other animal‐derived ingredients. The ddPCR method successfully identified porcine DNA in six out of the seven products, whereas the RT‐PCR method detected it in only three. This demonstrated the superior sensitivity of ddPCR in authenticating gelatin in commercial products. Furthermore, dietary supplement samples were confirmed to be free of porcine gelatin based on both ddPCR and RT‐PCR analyses. In a study by Sultana et al. [11], instances of mislabeling were identified in two collagen products labeled as fish gelatin, which were found to contain beef gelatin, as confirmed by RT‐PCR analysis with Ct values of 30.22 ± 0.12 and 29.34 ± 0.14, respectively. Additionally, mislabeling was detected in jelly beans and Yupi Gummi Pizza products, which contained pork gelatin, with Ct values of 30.12 ± 0.22 and 28.00 ± 0.20, respectively. Mahamad et al. [5] have reported the use of ddPCR to quantify and detect porcine and bovine DNA exclusively in gelatin capsule shells, revealing that 17 out of 55 capsules contained bovine and/or porcine DNA. In contrast, our method successfully applied ddPCR to both raw gelatin powder and commercial gelatin‐based confectionery products, demonstrating broader applicability across diverse sample matrices.

**Table 4:** Robustness test results on commercially processed products.

**Commercial product**	**ddPCR**	**RT-PCR**
	**Average copy number (copy/reaction)**	**Average Cq value**
Soft candy	6.20 ± 0.04	NA
Milk candy	3.10 ± 0.05	34.92 ± 0.68
Gummy candy	2.30 ± 0.16	NA
Marshmallow	2.40 ± 0.08	36.92 ± 1.10
Dietary supplement	0.00	NA
Capsule shell	1.20 ± 0.00	37.37 ± 0.60
Collagen cosmetic	4.00 ± 0.28	NA

### 3.5. Amplicon Recovery Analysis

This analysis was aimed at verifying that the amplified DNA was porcine, based on forward and reverse oligonucleotide sequences designed according to ISO/TS 20224‐3:2020(E). Sequencing of ddPCR amplicons from gelatin using the same primers validated the ddPCR method, as all droplets counted as positive were confirmed to contain porcine DNA. Figure 4 illustrates that the sequencing results of the confirmed amplicons exhibited high similarity to the reference species (*Sus scrofa*). Analysis with BioEdit showed significant similarity between the target sequence and the sequencing results (Figure 4a). Additionally, with NCBI, analysis indicated a 96% match with the target sequence (Figure 4b). These findings demonstrated that ddPCR was capable of accurately recovering and amplifying the target DNA.

Figure 4Results of amplicon recovery analysis using (a) BioEdit and (b) NCBI.

**Figure:**
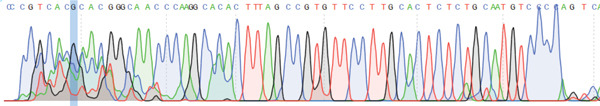
(a)

**Figure:**
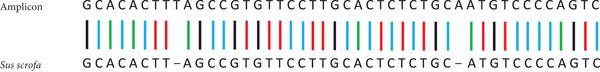
(b)

Amplicon recovery analysis was conducted to mitigate the risk of false‐positive results, which could have arisen from the presence of target DNA at levels below the detection limit of the ddPCR method, nonspecific amplification of background DNA that could have obscured the target DNA, and partial inhibition of DNA polymerase [23]. Sequencing of recovered amplicons allowed for the rapid and accurate reading of many targeted specific marker gene sequences [24].

## 4. Conclusion

This study successfully developed a ddPCR assay for the precise identification and quantification of porcine DNA in commercial gelatin products. The method demonstrated high specificity and sensitivity, detecting porcine DNA at concentrations as low as 0.06 ng/*μ*L, which surpassed the performance of conventional real‐time PCR. Validation across a diverse range of commercial gelatin products, including candies, marshmallows, dietary supplements, collagen, and capsule shells, confirmed the robustness and reliability of the ddPCR approach. Sequencing of ddPCR amplicons further verified that the detected DNA was porcine, showing high similarity to *Sus scrofa*. These findings underscore the potential of ddPCR as a valuable tool for routine halal detection, ensuring the integrity and compliance of gelatin products in the market.

## Conflicts of Interest

The authors declare no conflicts of interest.

## Funding

The author would like to express gratitude to the Ministries of Education, Culture, Research, and Technology of the Republic of Indonesia for their support of this research through the PTM grant (No. 3834/IT3.L1/PT.01.03/P/B/2022).

## Data Availability

Previously published research data, including articles, case studies, and reports, were utilized to support this study and are available in the sources listed in the reference section. All prior studies and datasets have been appropriately cited in the relevant sections of this manuscript as References [1–24]. The data that support the findings of this study are available on request from the corresponding author.
